# On the Management of Ventricular Arrhythmias Following Leadless Pacemaker Implantation

**DOI:** 10.19102/icrm.2021.121106

**Published:** 2021-11-15

**Keywords:** Leadless pacing, Micra, ventricular arrhythmia

## Drs. Blank and El-Chami comment

Ahmad et al.^1^ present an interesting case of a patient undergoing leadless pacemaker placement with subsequent ventricular fibrillation (VF) induced cardiopulmonary arrest. As they mention, there are several published case reports of ventricular tachycardia (VT) and VF proximate to implantation of the Micra™ device (Medtronic, Minneapolis, MN, USA). In their clinical scenario, a leadless pacemaker appears to have been the ideal device choice given the presence of complete heart block in a patient with end-stage renal disease on hemodiaysis, *Staphylococcous aureus* bacteremia, suspected endocarditis, and limited upper-extremity access. All reported device parameters that were observed upon implant appear to be acceptable but not ideal, and single-view X-ray suggested appropriate device positioning. A few concerning electrical parameters included an impedance of less than 800 Ω, which has been demonstrated to be an independent predictor of elevated Micra™ thresholds at 12 months,^2^ and an initial threshold of 1.3 mV, which is also a predictor of elevated thresholds during follow-up.^3^ These suboptimal numbers, while acceptable, could imply that the Micra™ device had suboptimal tissue contact.

Without any other clear inciting cause, the patient developed VF five hours after Micra™ implantation. The postarrest workup, including transthoracic echocardiography, was appropriate given the known risk for pericardial effusion and tamponade post–Micra™ implantation. While the rate of cardiac perforation associated with leadless pacing systems has been found to be less than 1% (0.77% in the Micra™ PAR population and 0.8% in the Micra™ CED population, respectively), this rate appears to be higher than that associated with transvenous pacemaker implantation (0.4% in the control group of the Micra™ CED study).^4^ In a report of the MAUDE database, three episodes of VF/VF were reported, all in the setting of perforation and effusion.^5^

While the authors state that the premature ventricular complex (PVC) that initiated the run of VF was of a different morphology that the paced QRS, it does appear in fact to be similar, with a rightward inferior axis and similar pre-cordial transition.

It is unclear whether the development of ventricular arrhythmia after leadless pacemaker implantation is due to the unique properties of the Micra™ fixation system (nitinol tines) or may be seen with all leadless pacemaker systems. While we have not seen this phenomenon in our clinical practice, this case report and others suggest that one might need to observe patients post–Micra™ implantation for 24 hours, calling our practice of same-day discharge into question.^6^ There are several potential explanations for the occurrence of VT/VF post–Micra™ implantation, as follows:

1. Poor tissue contact between the Micra™ and the right ventricular myocardium. If only two tines are engaged with the myocardium and are on the same side rather than opposite sides of the device, the Micra™ device could oscillate sideways and trigger early coupled PVCs, which could lead to VF.
2. There are some case reports^7^ of the Micra™ tines compressing a left anterior descending artery branch, which could lead to ischemia and VT/VF.
3. In patients with structural heart disease, right ventricular irritation could potentially lead to VT/VF.

When this occurs, the main treatment approach will require retrieval of the Micra™ and possible placement of a transvenous pacemaker or another Micra™ device in a different location. While there are no specialized tools available for Micra™ explantation, successful case reports documenting the use of standard sheaths and snares to remove the Micra™ device have been published.^8^ A recent report of outcomes comparing transvenous pacemakers and the Micra™ device as part of the Micra™ CED study did not compare rates of ventricular arrhythmias after implantation.^3^

We agree that further study of ventricular arrhythmias occurring after leadless pacemaker implantation require additional exploration and elucidation of the mechanism.

Evan Blank, md,^1^ and Mikhael El-Chami, md (melcham@emory.edu)^1^

^1^Emory University School of Medicine, Atlanta, GA, USA

Dr. El-Chami reports the receipt of consulting fees from Medtronic and Biosense Webster. Dr. Black reports no conflicts of interest for the published content.

References1.AhmadHBrarVButtNChetramVWorleySJO’DonoghueSVentricular fibrillation cardiopulmonary arrest following Micra™ leadless pacemaker implantationJ Innov Cardiac Rhythm Manage202112114756476010.19102/icrm.2021.121102PMC8631373348586682.KianiSWalaceKStrombergKA predictive model for the long-term electrical performance of a leadless transcatheter pacemakerJACC Clin Electrophysiol202174502512[CrossRef]
[PubMed]3335866610.1016/j.jacep.2020.09.0103.TolosanaJMGuaschESan AntonioRVery high pacing thresholds during long-term follow-up predicted by a combination of implant pacing threshold and impedance in leadless transcatheter pacemakersJ Cardiovasc Electrophysiol2020314868874[CrossRef]
[PubMed]3196736710.1111/jce.143604.PicciniJPEl-ChamiMWherryKContemporaneous comparison of outcomes among patients implanted with a leadless vs transvenous single-chamber ventricular pacemakerJAMA Cardiol202161011871195[CrossRef]
[PubMed]3431938310.1001/jamacardio.2021.2621PMC83198245.HauserRGGornickCCAbdelhadiRHTangCYCaseySASenguptaJDMajor adverse clinical events associated with implantation of a leadless intracardiac pacemakerHeart Rhythm202118711321139[CrossRef]
[PubMed]3371385610.1016/j.hrthm.2021.03.0156.KianiSBlackGBRaoBThe safety and feasibility of same-day discharge after implantation of MICRA transcatheter leadless pacemaker systemJ Atr Fibrillation20191212153[CrossRef]
[PubMed]3168706610.4022/jafib.2153PMC68113387.NguyenVIskandarMFothCRutzen-LopezHPattonMAlonsoRMicra pacemaker, macro complication. First reported case of coronary cameral fistula caused by leadless pacemaker implantJ Am Coll Cardiol20217718_Supplement_120968.AfzalMRDaoudEGCunnaneRTechniques for successful early retrieval of the Micra transcatheter pacing system: a worldwide experienceHeart Rhythm2018156841846[CrossRef]
[PubMed]2942782010.1016/j.hrthm.2018.02.008

## Dr. Orlov considers

VF following implantation of a leadless pacemaker is a rare complication; I have to admit that I have not seen this to date in our clinical practice. Proarrhythmic effects of cardiac rhythm management devices with leads, and cardiac resynchronization therapy in particular, have been reported by our group in the past.^1^ Details of the case presented by Ahmad et al.^2^ are fascinating, and several aspects require a specific comment. The patient’s initial electrocardiogram not only shows sinus rhythm but also a 2:1 atrioventricular block and Wenckebach periodicity, as well as significant QT prolongation. The corrected QT interval is not reported but seems to be prolonged to approximately 589 ms. It is unclear why this was present and whether QT prolongation could have contributed to the subsequent arrhythmic events. Additionally, the patient had end-stage renal disease, raising the possibility of causative electrolyte disturbances (although they seem to have been excluded by testing). It is also unclear whether the patient was paced at faster rates following device implantation to avoid bradycardia and polymorphic VT in the setting of a prolonged QT interval. Could this observation explain the subsequent ventricular arrhythmias and difficulty in resuscitating the patient? The authors’ **Figure 2** demonstrates right ventricular pacing immediately prior to the initiation of polymorphic VT, suggesting that the patient was indeed paced consistently.

The initial electrocardiogram during pacing is suggestive of the Micra™ device location being close to the outflow tract, which is frequently the case. The PVC morphology is not exactly the same, but very similar. I actually disagree that the triggering PVC is much different from the preceding right ventricular pacing morphology; instead, it seems to be a 6/7 leads match. Additionally, the Micra™ device can probably irritate nearby sites and not only the implant location due to its physical shape and length. Therefore, an inexact electrocardiogram match may represent mechanical irritation by the Micra™ device of a nearby site, resulting in a PVC and triggering ventricular arrhythmia.

The site of stimulation matters, and there are several observations to support this. In an animal model, high-frequency stimulation of the outflow tract was shown to be proarrhythmic and capable of causing outflow tract PVCs and ventricular arrhythmias.^3^ Arrhythmia inducibility was successfully suppressed by esmolol.^3^ Additionally, there is sufficient literature to support proarrhythmic effects of both left ventricular epicardial as well as right ventricular endocardial pacing during biventricular stimulation. In several cases, the arrhythmia was only abolished by discontinuation or modification of pacing (both left and/or right ventricular).^1,4^ Some cases have been successfully managed with ablation, frequently close to the stimulation site^5,6^ or pharmacologically with antiarrhythmic drugs, β-blockers, and/or steroids.^1^ Closer proximity of the pacing site to the putative reentry circuit is known to be proarrhythmic as demonstrated by pacing protocols from the right and left ventricular pacing sites.^7^ Right ventricular pacing was implicated as being proarrhythmic in several reports, including large database observations.^8–11^ Therefore, site-specific stimulation is likely to be proarrhythmic in some rare cases. These considerations are more pertinent to cases of scar-related VT where the reentry location is more defined. This does not seem to be the case in the case under discussion as no obvious scar substrate was present. However, Micra™ device location and proarrhythmia, either due to the proximity to the arrhythmogenic area or right ventricular pacing itself, seem to be the likely cause (in the setting of prior QT prolongation, possibly). It is unclear whether leadless device repositioning could be utilized as a strategy in the future if similar complications occur. Certainly, during the implantation procedure, particularly while the device is still tethered, repositioning in case of a sustained ventricular arrhythmia could be considered. Of note, the Postapproval Micra™ Registry has not reported ventricular arrhythmias as a complication, while smaller studies did (see discussion of the case report by Ahmad et al.). The role of antiarrhythmic drugs and β-blockers that have been reported to mitigate lead-related proarrhythmia in some cases remains to be determined.

It seems that leadless pacemakers are safe and associated with few side effects, possibly less than cardiac rhythm management devices with leads. Certainly, some specific lead-related complications will not occur with leadless devices. Other complications may be much more likely to occur with leadless devices (for example, retrieval of a dislodged device) compared to leads. This specific Micra™ case highlights the importance of vigilance and possibly more extensive monitoring immediately after leadless device implantation. At the very minimum, same-day discharge may seem less attractive after this and similar case reports. Additionally, physiologic pacing, which was successfully used to mitigate the proarrhythmic effects of right ventricular pacing,^12^ is being increasingly utilized and may be less likely to result in life-threatening ventricular arrhythmias.

Michael V. Orlov, MD, PhD, FACC (michael.orlov@steward.org)^1,2^

^1^Steward St. Elizabeth’s Medical Center of Boston, Boston, MA, USA

^2^Tufts University School of Medicine, Boston, MA, USA

The author reports no conflicts of interest for the published content.

References1.ShuklaGChaudhryGMOrlovMHoffmeisterPHaffajeeCPotential proarrhythmic effect of biventricular pacing: fact or myth?Heart Rhythm200529951956[CrossRef]
[PubMed]1617174910.1016/j.hrthm.2005.05.0192.AhmadHBrarVButtNChetramVWorleySJO’DonoghueSVentricular fibrillation cardiopulmonary arrest following Micra™ leadless pacemaker implantationJ Innov Cardiac Rhythm Manage202112114756476010.19102/icrm.2021.121102PMC8631373348586683.ZhouJScherlagNJYamanashiWExperimental model simulating right ventricular outflow tract tachycardia: a novel technique to initiate RVOT-VTJ Cardiovasc Electrophysiol2006177771775[CrossRef]
[PubMed]1683667610.1111/j.1540-8167.2006.00509.x4.MiśkowiecDŻycińskiPQawoqDHRight ventricular lead induced ventricular arrhythmia—a rare complication of cardiac resynchronization therapyAnn Noninvasive Electrocardiol2019245e12666[CrossRef]
[PubMed]3124124110.1111/anec.12666PMC69318885.PeichlPMlcochováHKautznerJSuccessful catheter ablation of two types of ventricular tachycardias triggered by cardiac resynchronization therapy: a case reportJ Cardiovasc Electrophysiol2007182218221[CrossRef]
[PubMed]1733877010.1111/j.1540-8167.2006.00676.x6.PedrettiSVargiuSPaolucciMLunatiMA case of premature ventricular contractions, ventricular tachycardia, and arrhythmic storm induced by right ventricular pacing during cardiac resynchronization therapy: Electrophysiological mechanism and catheter ablationJ Arrhythm2015316401405[CrossRef]
[PubMed]2670232410.1016/j.joa.2015.06.002PMC46720787.RobertsonJFCainMEHorowitzLNAnatomic and electrophysiologic correlates of ventricular tachycardia requiring left ventricular stimulationAm J Cardiol1981482263268[CrossRef]
[PubMed]727043510.1016/0002-9149(81)90606-88.CroninEMJonesPSethMCVarmaNRight ventricular pacing increases risk of appropriate implantable cardioverter-defibrillator shocks asymmetrically: an analysis of the ALTITUDE databaseCirc Arrhythm Electrophysiol20171010e004711[CrossRef]
[PubMed]2903037910.1161/CIRCEP.116.0047119.BelhassenBKerenGOstrzegaECoppermanILaniadoSVentricular tachycardia induced by slow ventricular pacingIsr J Med Sci1984201211771182
[PubMed]
651995010.TaniguchiHUnoKKomatsuYIesakaYAblation of epicardial right ventricular tachycardia provoked by proarrhythmic RV pacing in a patient with dilated cardiomyopathyPacing Clin Electrophysiol2012355e140e143[CrossRef]
[PubMed]2171479110.1111/j.1540-8159.2011.03144.x11.ArcinasLAMcIntyreWFFaragAKushneriukDHiebertBSeiferCMRight ventricular pacing is associated with increased rates of appropriate implantable cardioverter defibrillator shocksAnn Noninvasive Electrocardiol2019244e126363071981910.1111/anec.12636PMC693162612.SofiAVijayaramanPBaroldSSHerwegBUtilization of permanent His-bundle pacing for management of proarrhythmia related to biventricular pacingPacing Clin Electrophysiol2017404451454[CrossRef]
[PubMed]2800030910.1111/pace.12997

## References

[1] Ahmad H, Brar V, Butt N, Chetram V, Worley SJ, O’Donoghue S (2021). Ventricular fibrillation cardiopulmonary arrest following Micra™ leadless pacemaker implantation. J Innov Cardiac Rhythm Manage.

[2] Kiani S, Walace K, Stromberg K (2021). A predictive model for the long-term electrical performance of a leadless transcatheter pacemaker. JACC Clin Electrophysiol.

[3] Tolosana JM, Guasch E, San Antonio R (2020). Very high pacing thresholds during long-term follow-up predicted by a combination of implant pacing threshold and impedance in leadless transcatheter pacemakers. J Cardiovasc Electrophysiol.

[4] Piccini JP, El-Chami M, Wherry K (2021). Contemporaneous comparison of outcomes among patients implanted with a leadless vs transvenous single-chamber ventricular pacemaker. JAMA Cardiol.

[5] Hauser RG, Gornick CC, Abdelhadi RH, Tang CY, Casey SA, Sengupta JD (2021). Major adverse clinical events associated with implantation of a leadless intracardiac pacemaker. Heart Rhythm.

[6] Kiani S, Black GB, Rao B (2019). The safety and feasibility of same-day discharge after implantation of MICRA transcatheter leadless pacemaker system. J Atr Fibrillation.

[7] Nguyen V, Iskandar M, Foth C, Rutzen-Lopez H, Patton M, Alonso R (2021). Micra pacemaker, macro complication. First reported case of coronary cameral fistula caused by leadless pacemaker implant. J Am Coll Cardiol.

[8] Afzal MR, Daoud EG, Cunnane R (2018). Techniques for successful early retrieval of the Micra transcatheter pacing system: a worldwide experience. Heart Rhythm.

